# Wireless Sensor Network in Agriculture: Model of Cyber Security

**DOI:** 10.3390/s20236747

**Published:** 2020-11-25

**Authors:** Radomir Prodanović, Dejan Rančić, Ivan Vulić, Nenad Zorić, Dušan Bogićević, Gordana Ostojić, Sohail Sarang, Stevan Stankovski

**Affiliations:** 1 Centre for Applied Mathematics and Electronics, Serbian Armed Forces, 11000 Belgrade, Serbia; 2 Faculty of Electronic Engineering, University of Niš, 18000 Niš, Serbia; dejan.rancic@elfak.ni.ac.rs; 3 Military Academy, University of Defence, 11000 Belgrade, Serbia; ivan.vulic@mod.gov.rs (I.V.); dusan.bogicevic@vs.rs (D.B.); 4 Faculty of Technical Sciences, University of Novi Sad, 21000 Novi Sad, Serbia; zoric.de17.2019@uns.ac.rs (N.Z.); goca@uns.ac.rs (G.O.); sohail@uns.ac.rs (S.S.); stevan@uns.ac.rs (S.S.)

**Keywords:** wireless sensor networks (WSN), agriculture, security, cryptography, public key infrastructure (PKI)

## Abstract

Nowadays, wireless sensor networks (WSN) are widely used in agriculture monitoring to improve the quality and productivity of farming. In this application, sensors gather different types of data (i.e., humidity, carbon dioxide level, and temperature) in real-time scenarios. Thus, data gathering, transmission, and rapid response to new circumstances require a secured data mechanism to avoid malicious adversaries. Therefore, this paper focuses on data security from the data origin source to the end-user, and proposes a general data security model that is independent of the network topology and structure, and can be widely used in the agriculture monitoring application. The developed model considers practical aspects, the architecture of the sensor node, as well as the necessity to save energy while ensuring data security, and optimize the model through the application of organizational and technical measures. The model evaluation is conducted through simulation in terms of energy consumption. The result shows that the proposed model ensures good data security at the cost of a slight increase in energy consumption at receiver and sender nodes, and energy consumption per bit, up to 2%, 7%, and 1.3%, respectively, due to overhead added for authentication in the network.

## 1. Introduction

Agriculture is one of the last, largest systems in the world that is not yet entirely digitalized. It is a biological production system with many factors affecting its complexity, for instance, human behavior, machines, nature, chemicals, biology, weather, and climate.

In such a complex system, it is necessary to gather and analyze different types of data in decision-making. There are many factors that affect data gatherings in agriculture monitoring, such as specific characteristics of the geographic location from where data is gathered, weather and climate, the proximity of markets and infrastructure for transportation and storage, agricultural methods, and activities individualized as the people performing them [1].

Nowadays, sensors are used for rapid real-time data gathering in agriculture using wireless sensor networks (WSNs). Data gathered in this way show immediate change enabling the system to monitor agri-parameters in real-time. Access to a large amount of data generated from different sources in a short time creates difficulties in making the best decision. Numerous data are processed by advanced analytical abilities [2,3].

The recent developments in agriculture do not depend only on the amount of different data gathered in different ways, but also on the security of data gathered. This is because security attacks, such as eavesdropping, disruption, physical, and others, can alter information and network structure. Thus, to ensure that data gathered by the sensor remain authenticated, unchanged, and protected, it is necessary to establish a security model from the source node (origin) to the receiver (destination) in the communication network and application for data processing and storing.

Therefore, a data security model is developed that provides data protection from sensors, to wireless networks and applications for data processing by encryption, digital envelopes, digital signature, and public key infrastructure (PKI). In addition, the model is independent from communications infrastructure, providing security from one point to another point, as well as its application in sensor networks in other aspects of human activities requiring the security of data gathered for management purposes. Furthermore, it fulfills the security requirements of a distributed system, such as authentication, authorization, data integrity, data trustworthiness, availability, non-repudiation, trust, and privacy.

The purpose of this paper is to develop a model of security sensor networks independent from communications infrastructure, providing security from one point to another point, as well as its application in sensor networks in other aspects of human activities requiring the security of data gathered for management purposes. The rest of the paper is organized as follows: the next section presents the basis of the wireless sensor networks, their use in agriculture, security requirements to be fulfilled, and existing wireless sensor network security solutions; section three describe applied materials and methods; section four describes a model for wireless sensor networks protection; section five provides simulation results and proposed model evaluation; section six presents an optimized model; and section seven provides a conclusion.

## 2. Related Work

Sensor Network (SNet) is a type of widely pervasive distributed system consisting of multiple sensor nodes deployed in a field that have the ability to mutually communicate via wireless networks [4]. The task of this distributed system is to gather data by sensors and identify information best describing the monitored phenomenon.

WSN is a wireless network consisting of spatially distributed unassisted devices, called sensors, for monitoring the physical or environmental conditions and parameters [4,5,6,7]. WSNs have become increasingly used in agriculture. They may be applied in the following areas [8]: environmental status monitoring [9,10,11], precise agriculture [12,13,14], machinery and processes control [15,16], automatization of monitored areas, and monitoring systems [17,18,19,20,21].

Sensor Network, as a type of distributed system, should fulfill the security requirements of distributed systems, such as [22]: authentication, authorization, data integrity, data trustworthiness, availability, non-repudiation, trust, and privacy.

Authentication is a process of confirmation of the identity of a participant communicating in a sensor network. Authorization is a process of verification if an entity (user, sensor, software) is entitled to perform a required or initiated action. Integrity means that data are not modified or destroyed in an unauthorized way. Data integrity guarantees that sensor data will not be modified in an unauthorized manner in the process of their transmission, processing, or archiving. Trustworthiness means that data are not available to, or at, the disposal of the unauthorized for the reception. Sensor networks protected data include indirect data, such as the ones accessed, based on transmission or monitoring (supervision) of transmission processes. Availability is a requirement securing the system’s prompt response, as well as the availability of services to all authorized users. ITU-T (International Telecommunication Union Telecommunication Standardization Sector) X.509 defines trust as: “Generally, one entity may say to “trust” the other entity when the first entity expects the second entity to behave as expected”. Non-repudiation is of vital importance in the process of enabling legitimate transactions that must be recognized and non-repudiated from all entities involved. Privacy is a more complex issue than trust and relates to securing user information or any other part of data from others, entirely or partially. Data privacy is any attempt at securing information, and is available only to those with authorized access.

### 2.1. Wireless Sensor Network Attacks

Attacks on the security of WSN may be divided into two wide categories [23]: passive and active attacks. A passive attack is the one when attackers make no emission, and it is mostly directed towards data trustworthiness. In active attacks, ill-will activities are focused, not only on data secrecy, but also on data integrity. In addition to security attacks, security is affected by users’ behavior, which may expose nodes to threats by an error, such as destroying, unauthorized access to secret data, and resources [24,25].

The WSN network communication architecture consists of layers. Layered architecture, with its vulnerabilities, allows various types of attacks that can be divided into the following:

- Physical layer attacks: physical attacks on WSNs range from node capturing to the jamming of the radio channel [26,27]. In this attack, the adversary attempts to disrupt the operation of the network by broadcasting a high-energy signal [28] to prevent sensor nodes from communicating.
- Data link layer attacks: the functionality of link-layer protocols is to coordinate neighboring nodes to access shared wireless channels and to provide link abstraction to upper layers. The attacker disrupts the operation of the protocol by causing a collision packet, inserting, and interrogating messages to obtain information on the communication template, or slow down communication [29,30,31].
- Network layer attacks: the network layer is susceptible to various types of attacks that are aimed at interrupting routing and functionality of the entire WSN. A characteristic attack is an attack on sync holes by directing all traffic to a compromised node that looks like a real node, but with an attacker in the center. Malicious or attack nodes do not route messages and, thus, prevent further forwarding of messages [27,28]. An attacker can deceive, modify, or reproduce routing information to disrupt traffic in the network.
- Transport layer attacks: the attacker can make as many connection requests as possible by exhausting all of the resources needed for each connection, or reaching the maximum limit. This attack creates significant limitations for legitimate nodes [25,30].
- Application layer attacks: different types of attacks can be implemented on the application layer, such as overwhelm, repudiation, data corruption, and the implementation of malicious code. This attack exhausts the network bandwidth and leads to the high-energy consumption of nodes [32].

Based on the way the attacker performs attack, attacks on the WSN can be classified into the following five categories: eavesdropping, traffic analysis, disruption, hijacking, and physical attack.

An eavesdropper tries to identify what data go out from the sensor network. The eavesdropper either listens to the messages transmitted by nodes or directly jeopardize nodes. Eavesdropping may take two forms. Passive eavesdropping occurs when the eavesdropper passively intercepts messages without discovering its presence. Active eavesdropper sends queries to the sensor or aggregation point or attacks sensor nodes in order to obtain more information.

By traffic analysis, the attacker may identify the pattern of the traffic network and WSN topology consequently. Information obtained by the traffic analysis helps the attacker discover location, weaknesses, functions, or node proprietors.

The aim of the attacker is to disrupt sensor application. In order for the attack to be as efficient as possible, the attacker is focused on sensor node gathering and aggregating data. The attack is performed by using two techniques. Semantic disruption is achieved by the introduction of false messages, data damaging, and change of values to make aggregate data compromised, useless, or incomplete. Physical disorder disrupts sensor readings directly by manipulating the sensor environment, e.g., by the creation of additional heat, humidity, or other circumstances surrounding sensors.

The hijacking attacker disrupts the exit of the sensor in order to obtain control over it. The attacker carefully selects a set of sensors combining eavesdropping and disruption within the sensor network. This type of attack is most difficult for handling as it originates from reliable nodes [24]. The attacker takes over communications between two nodes and establishes communications with them. The attacker deceives nodes by false data.

The attacker physically damages the sensor hardware and disables its operations. The physical attack may be considered as error tolerance, meaning it can maintain the network functionality with no disruptions due to node malfunction.

Attacks on wireless sensor networks are many and are related to the following aspects [33]: sensor theft, attacks on software and communications protocols, attacks on protocols for key management, attacks on sensor scheme, attacks on network data processing, and attacks on time synchronization protocols.

Taxonomies of attack are very useful for designing security mechanisms. There are several taxonomies for wireless networks [34] and taxonomies for WSN [35,36,37]. Taxonomies classify attacks based on the purpose, behavior, and goal of the attack, helping to better understand the principle of attacking the WSN and designing better countermeasures for sensor networks.

### 2.2. Wireless Sensor Network Security Solutions

In [38], the authors study the problem of authentication to prevent unauthorized access to the WSN for agriculture. They propose remote user identity verification and a key-agreement protocol for agriculture monitoring in WSNs. The proposed scheme is applicable in real time, but it is too complex.

In paper [39], the proposed scheme consists of six phases: system setup phase; user/agriculture professional registration phase; login phase; authentication and session key agreement phase; password update or change phase; and dynamic node addition phase. The proposed scheme represents a significant improvement of Ali et al.’s scheme [38]. Ali et al.’s scheme consists of four entities instead: the user/agriculture professional, base station, sensor node, and gateway node. In this scheme, authentication and key agreement depend on the central entities (base stations). Without base stations, entities will not be able to trust each other.

In paper [40], the authors point to the problem of authentication in order to prevent unauthorized use of sensors and resources in sensitive jobs. The authors present an authentication based on Burrows–Abadi–Needham (BAN) logic and formal security analysis for coal mines in WSNs. BAN logic ensures the mutual authentication and session key agreement properties. The formal security analysis proves its resilience against various security attacks. Applying authentication to underground mines significantly reduces the possibility of loss of resources and people. The proposed authentication protocol provides strong authentication and raises the level of security.

Turkanović et al. [41] proposed a novel user authentication and key agreement scheme for heterogeneous ad hoc wireless sensor networks. The proposed scheme enables a remote user to securely negotiate a session key with a general sensor node, using a lightweight key agreement protocol. The proposed scheme ensures mutual authentication between the user, sensor node, and the gateway node (GWN), although the GWN is never contacted by the user. The proposed scheme has been adapted to the resource-constrained architecture of the WSN; thus, it uses only simple hash and XOR (exclusive disjunction) computations.

Amin and Biswas [42] designed a novel architecture for the WSN environment, based upon which a proposed scheme has been presented for user authentication and a key agreement scheme. This scheme fixes the above-mentioned security pitfalls (Turkanovic et al.’s scheme). The security validation of the proposed protocol was done by using BAN logic, which ensures that the protocol achieves mutual authentication and session key agreement property securely between the entities involved. The proposed protocol not only resists the above mentioned security weaknesses, but also achieves complete security requirements, including (especially) energy efficiency, user anonymity, mutual authentication, and a user-friendly password change phase.

Yeh et al. [43] proposed protocol that can prevent all of the problems of the former schemes [44,45,46] and provide mutual authentication to protect inside security and outside security. Furthermore, it not only inherits the merits of ECC (Elliptic-curve cryptography)-based mechanism, but also enhances the WSN authentication with higher security than other protocols.

Recent study by Dos Santos [47], in 2019, resulted in the Agri prediction model that was experimentally studied and developed. He implemented WSN with a prediction model, which combines the ARIMA (Auto-Regressive Integrated Moving Average) prediction model with Long Range Wide Area Network (LoRaWAN) technology (short and medium area coverage) in monitoring of the arugula crop. Developed WSN is collecting data of the form land information and notifying farmers of when to plant the arugula. This work presents successful implementation of LoRa technology in agriculture monitoring with the prediction model, which warns the farmers about abnormal situations in advance. The main drawback of the proposed model is the real-time monitoring application, which should be developed in future work with a mobile application.

Report of Naoui et al. [48] provided a new security mechanism for LoRaWAN [47]. In this paper, the LoRaWAN gateway was extended to the concept of a proxy node. Basically, the proxy nodes act as the gate, which evaluates data flow trustworthiness from sensor nodes to the end node. Apparently, the proxy node becomes the place of the highest trust value—what can be a serious drawback if an attacker targets that node. In general, the common issues for LoRaWAN security models are key updates and session key generation.

## 3. Materials and Methods

The objective of this research is to create a model for WSN protection in agriculture on the sensor level that will enable the protection of collected information, as well as the authentication of sensors in communication. In order to achieve this objective, we researched WSN security literature, identified the most common attacks on WSN, and defined the security requirements for WSN. We perceived implementation of WSN in the agriculture environment, researched, and applied the symmetric and asymmetric cryptography and public key infrastructure for model development.

For research, literature available in scholarly databases was used, such as: ABI/INFORM Global, Academic Search Premier, ACM Digital Library, Applied Science and Technology Full Text (EBSCO), IEEE Xplore, ScienceDirect and Google Scholar.

After considering the implementation of WSN in agriculture [8,9,10,11,12,13,14,15,16,17,18,19,20,21] as a distributed system, we researched attacks on the WSN [23,24,25,34,35,36,37]. Attacks on WSN exploit vulnerabilities in all layers of network communication architecture, while disrupting security requirements, such as authentication, authorization, data integrity, data trustworthiness, availability, non-repudiation, trust, and privacy [25,26,27,28,29,30,31,32]. In order to meet WSN security requirements in the model, we applied cryptography-based security technologies.

WSN for monitoring of agriculture conditions is designed with the Flora framework of OMNeT++ software. We evaluate the performance of the proposed model in terms of energy consumption at the receiver, energy consumption per bit, and sender node using the Castalia 3.3 simulator.

## 4. Proposed Model of Security Wireless Sensor Network in Agriculture

The model is designed to fulfill the above-mentioned security requirements of sensor networks. The general principle of the model is to securely identify communications actors and transmit data, enabling non-repudiation of message reception, as well as facilitating the availability of reliable data for decision-making. The modal is based on the protection of data exchanged by encryption, digital envelope, digital signature, and PKI within the sensor network attacks.

### 4.1. Security Technologies Applied in the Model

Encryption enables the transformation of data in a form that only the one for whom it is intended may read it. A symmetric cryptography for generating the secret key and message encryption is used in the model. Asymmetric cryptography and PKI digital certificates are used for digital signing of messages and protection of the secret key.

The technology of digital envelope is used in the model for the message and secret key encryption. In this way, only the message receiver may have an insight into the message content. With a digital envelope, the message content is firstly encrypted by a specific symmetric algorithm (e.g., DES (Data Encryption Standard), 3-DES (Triple DES), RC2 (Rivest Cipher), AES (Advanced Encryption Standard), or by a specific private algorithm). Then, a secret key is encrypted by an asymmetric cryptographic algorithm (e.g., RSA (Rivest–Shamir–Adleman) algorithm) of the public key from the digital certificate of the receiver. On the side of the message reception, the secret key is decrypted by a private key of the receiver, and then the message is decrypted by a received secret key.

Digital signature signs the message by firstly reducing the message into a message digest, and then, the received digest is encrypted by an asymmetric cryptographic algorithm, for example, RSA algorithm by using the private key of the message signatory. The digital signature becomes an extended part of the message. The receiver completes the process of verification of the digital signature.

The purpose of the certificate is to establish a link between an identified (notified) entity and the public key, indirectly with the corresponding private key of the entity. This is accomplished when the Certification Authority uses its private key for signing the certificate, so that the certificate can later be verified by any entity, which has the public key of the Certification Authority.

### 4.2. Wireless Sensor Network in Agriculture Protection Model Proposed

**Figure 1:**
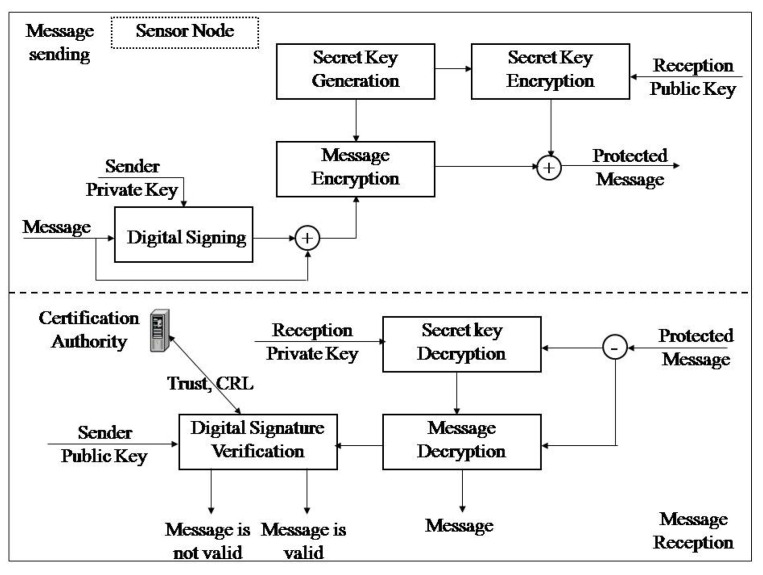
Wireless Sensor Network in Agriculture Protection Model.

The model secures the fulfillment of security requirements of the wireless sensor network in the process of exchange of data gathered and management messages between two sides of a wireless sensor network by cryptography and PKI. The sender generates a secret key to encrypt the message by asymmetric algorithm. Digital signing of the message is done by asymmetric cryptography, as well as protection of the secret key in the process of transmission to the receiver. The receiver decrypts the encrypted secret key by asymmetric cryptography and then decrypts the encrypted message using the secret key. The receiver conducts verification of the digital signature in order to verify the integrity and the message sender. Secure distribution of the secret key and establishment of a unique connection between the user and the public key is done by PKI application. The Wireless Sensor Network in Agriculture Protection Model Proposal is given in Figure 1.

#### 4.2.1. Preparation for Model Implementation

Preparation for the model implementation in the sensor network includes the preparation of the configuration and configuration of the sensor networks.

In the process of preparation for configuration, sensor network risk evaluation is conducted to select the appropriate symmetric algorithm and duration of the secret key for message protection. Users may use the existing public symmetric algorithm or buy specially developed ones. Moreover, it is necessary to select a certification body to issue digital certificates for sensor networks for digital signature and secret key distribution. PKI, with a certification body, may be established by the user for its own purposes.

Sensor network configuration includes the introduction of selected crypto-algorithms, crypto-keys, and digital certificates into sensor nodes, nodes for data processing and aggregation, as well as into the application for processing and management of the sensor network.

#### 4.2.2. Protection of the Sensor Node Message in the Process of Transmission

**Figure 2:**
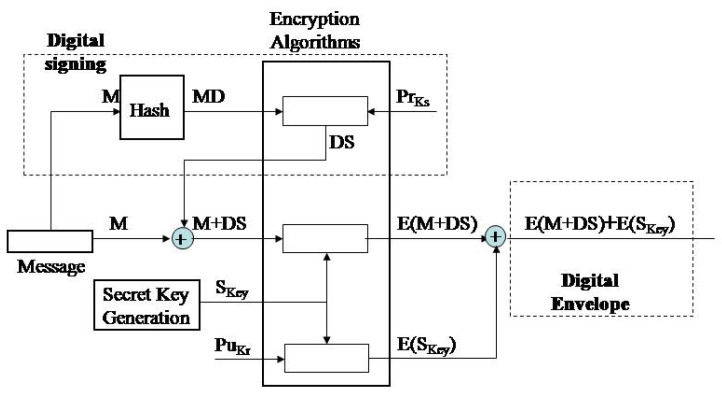
Detailed overview of the message protection in the transmission process.

Upon gathering data by the sensor node, data aggregation into a message is performed. The message is reduced into a message digest (MD) by application of some of the message-digest algorithms (MD5, SHA-1 (Secure Hash Algorithm), SHA-256). This message digest is encrypted by asymmetric cryptographic algorithm (e.g., RSA) by use of the private key of the message signatory, key PrKs, Figure 2. The encrypted message digest is a digital signature of the given message (DS) and becomes her enclosed part. Upon this, a secret key (SKey) is generated for encryption of the message and digital signature by symmetric cryptographic algorithm. Then, the secret key, SKey, is encrypted by a public key of the receiver, PuKr, and merges with the previously encrypted message E (M + DS).

Prior to the digital signing of the message, the sender performs verification of the trust chain and status for the digital certificate of the receiver, as well as the status of its own digital certificate. Check of the trust chain identifies if the trusted certification body issued a certificate, meaning if the receiver may be trusted. If there is no trust, the transmission process is interrupted, as there is a false receiver. The status of digital certificate check is performed by a list of revoked certificates or OCSP (Online Certificate Status Protocol) inquiry, depending on the implementation of the certificate status check. If revoked, the digital certificate proves that the sensor or application cannot be trusted anymore. The sensor node is not trustworthy if compromised or if it is confirmed that it provides false data. In this case, the communication is aborted. If the digital certificate is suspended, it means that sensor node data are temporarily unnecessary. On the other hand, in the process of protection of the secret key of the sender, a check of the digital certificate of the receiver is performed. If determined that the certificate of the receiver is revoked or suspended, this means that the receiver (server, data processing application, data aggregator) is compromised or currently unavailable. In this case, the entire process of sensor node data is aborted.

#### 4.2.3. Verification of the Content of the Protected Message in the Process of Reception

**Figure 3:**
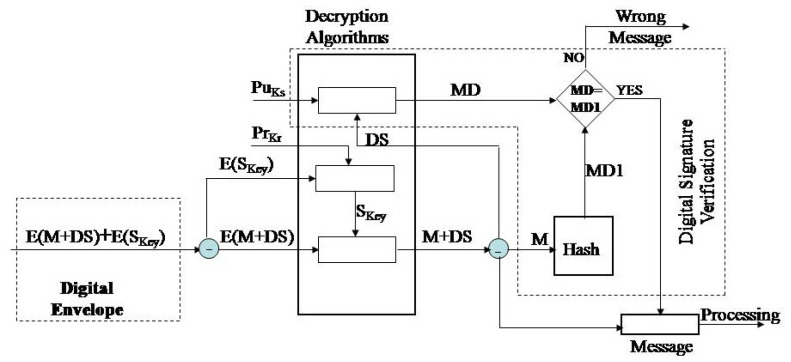
Detailed overview of the message verification phase.

In the reception phase, Figure 3, the receiver decrypts the secret key and verifies the digital signature. The decryption of the secret key is performed by the private key of the message receiver, the key PrKr. The message E is decrypted by the obtained secret key (M + DS). Verification of the digital signature consists of decryption of the message digest by asymmetric algorithm and application of the public key of the message sender, the key PuKs. Upon decryption of the digital signature, the message receiver performs the same message digest procedure of the received message, M. If the message digest, MD1, is identical with the decrypted digest value, MD, the verification is considered successful; if not, then the verification was unsuccessful.

If the verification was successful, the message receiver is certain about the authenticity of the sender, the integrity of the message sent, and the inability of the sender to subsequently revoke message sending.

In this phase, the verification of the trust chain is performed, as well as of the status of digital certificates of the sender and the receiver. The trust chain is verified for the certificate of the sender. If there is no trust in the chain of certificates of the sender, then the message reception is aborted as data are probably sent by the sensor node introduced by the attacker. If the status of the digital certificate of the sensor node of the sender is revoked or suspended, it means that the receiver has no trust in the data received. Similarly, if the status of the digital certificate of the receiver is revoked or suspended, it means that there is no longer trust in the public and private keys of the receiver. This leads to a lack of trust in the secure transmission of the secret key.

### 4.3. Characteristics of the Model

Fulfillment of security requirements for distributive systems. The security requirement of authentication is met by the application of the digital signature. Every node is allocated a digital certificate signed by the authority (certification authority) trusted by all users if the sensor network. Verification of the digital signature by public key from the digital certificate of the sender confirms the identity of the sender’s node and message integrity. Non-repudiation is realized by the inability of the sender to subsequently revoke message sending if his digital signature is successfully verified. The trustworthiness of data is achieved by message encryption by symmetric cryptographic algorithms, while secrecy of the key is realized by encryption with asymmetric cryptographic algorithms. In this model, trust is built by the certification body trusted by all users of the sensor network, as it issues a digital certificate to all. The availability of secure data in further processing is achieved by authenticity, trustworthiness, and message integrity in the process of transmission. Privacy is achieved by PKI application itself, in the process of users’ registration (sensor nodes, aggregation nodes, applications, individuals) and digital certificate issuance.

Wireless sensor network data management. The model enables the management of data gathered by sensor nodes. Management is conducted via the digital certificate unique for every sensor node by making digital certificate status revoked or suspended. If in the process of verification of the digital certificate status of the sender or receiver, proves that the status is revoked or suspended, message sending is aborted. In this case, data may be sent to the aggregation node or to the application for data processing, which will subsequently perform data analysis depending on the digital certificate revocation or suspension reasons. The model enables that depending on the reason for certificate status change, data may be treated differently.

Secure distribution of the secret key in a wireless sensor network. The model enables the secure transmission of the secret key to every sensor node. This characteristic may be used to lessen the node processor burden in the process of data protection when sending a message. The model may be modified so that in the process of initialization, the same secret key may be implemented in all nodes, while the new secret key is distributed to all nodes in case of being compromised or periodic renewal.

Message protection from point to point. This characteristic provides message protection from the data source to the end destination, even in the case the message is transmitted via several nodes. In the process of transmission of the protected message via several nodes, it is not possible to have insight into the message content. This enables the receiver to be certain that the message secrecy is not compromised in the transmission process and that data originates from the authentic sender (source node). In the case of merging data in the aggregation node and its further distribution, it is necessary to keep the digital message signature. In this case, the receiver of data may be sure that the original data has not to be modified and that it originates from the source node.

The model is not dependent on the wireless sensor network applied in agriculture architecture. This means it may be applied in different architectures of the sensor networks in agriculture, such as architectures based on the changed positions of sensor nodes (stationary architecture, mobile architecture, and hybrid architecture), different categories of architecture based on hierarchy (single-tier architecture, multi-tier architecture). Additionally, our model is based on data protection from the sensor node to the application where gathered data are processed, as from the management application to sensors. This means that any single movement, flow, or rest of the sensor node, as well as its position in the hierarchy-based architecture will not affect the model application.

In regard to different communication technologies, the proposed model is not dependent on the application of relevant technologies in the sensor network applied in agriculture, such as ZigBee, Wi-Fi, Bluetooth, GPRS (General Packet Radio Service)/3G/4G and WiMAX (Worldwide Interoperability for Microwave Access). More specifically, such an approach does not require the application of any specific technology for wireless transfer as the processes of the model are performed prior to sending the message, as well as after the message is received.

Considering implementation of new operations, the model creates an additional burden to the hardware and software architecture of nodes. This will affect the necessary modifications of the existing wireless platforms of the sensor nodes used in agriculture. It is necessary to modify middleware and sensor from the category—soil, environment, and plant-related by incorporation of the crypto-modules.

### 4.4. Case Study Example for the Application of the Model in the Irrigation System 

The field irrigation system consists of sensors (heat, soil humidity, and nutrition), nods for data gathering (gateway nods), and aggregation (sink node), an irrigation system management unit (two sensors—actuators for water flow and nutrients intake control) and a control center for management of the system. The system has the purpose to enable the best conditions for cultivation by gathering data on temperature, soil, and humidity, and recommends the best quantity of nutrients and water. There is an experimental field. Competitors are interested in eavesdropping the wireless sensor network during the experiment in order to gather data without authorization, or to change them so that the experiment results in the wrong data.

In order to prevent competition from data gathering of receiving management messages of the wireless sensor network, the sensor with crypto-modules and the proposed optimized model will be applied.

**Figure 4:**
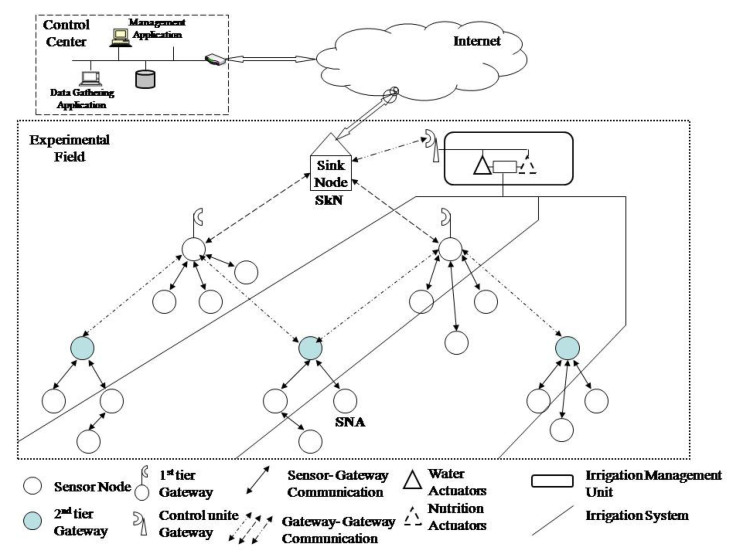
The architecture of the observed experimental field.

The sensor network gathers data on agri-parameters and transmits the via gateway sensor to the sink node (SkN). The sink node transmits data via the internet to the internal network of the control experimental center where data processing is performed, and makes a decision on irrigation and necessary nutrients, Figure 4.

Prior to the implementation of the sensor network, phases for the protection model based on PKI are implemented. In the first phase, assessment of the data security risk was conducted and it was identified that there was a risk existing from the insight into the message content, data modification, as well as from replacement of the sensor nodes by nodes providing false data. Security risks from data change exist not only in the wireless sensor network but in the process of data transmission via the internet, too. Table 1 provides parameters for the initialization of the model.

In the second phase, a selected cryptographic algorithm is implemented into sensor nodes, actuator nodes, sink nodes, as well as digital signature software, digital certificates, and private key of nodes. Moreover, selected crypto-algorithm digital certificates, cryptographic keys, digital signature, and its verification software are implemented in the computers for the data gathering application and decision-making application.

Using a data gathering application, the control center transmits the message to sensor nodes requiring them to send data gathered. The message for data gathering is transmitted via an internet network and wireless sensor networks. Communications in the wireless sensor network may be organized in such a way that the sink node performs some of the data processing and aggregation, and then transmit them to the data gathering application. The other way is if the sink node only aggregates data of sensor nodes and transmits them to the data gathering application.

**Figure 5:**
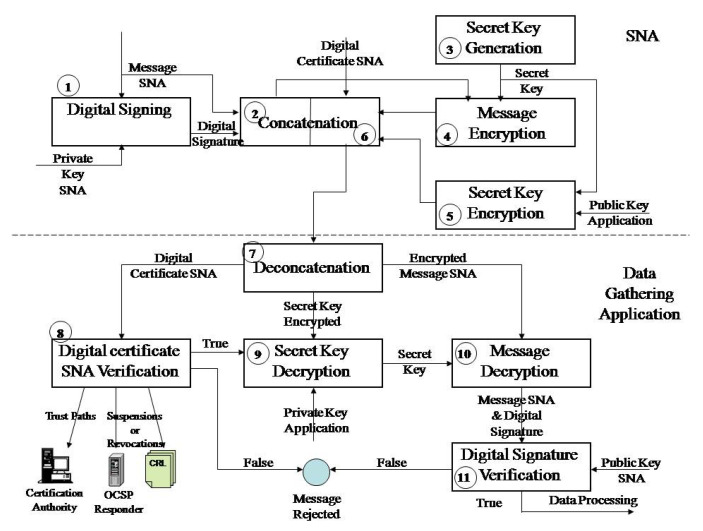
Diagram of data protection phase and message content verification phase.

Assuming that the SNA node sends data gathered from the experimental field to the data processing application, and that SkN does not process, but only forwards data, in this case, firstly, the SNA node performs digital signing of the message by the private key of the node (1), Figure 5. Then, the aggregation of digital signature and message is performed (2). SNA node generates the secret key (3) that uses a symmetric algorithm to encrypt the message and digital signature (4). Then, the asymmetric cryptographic algorithm with the application of the public key of the node of the data gathering application encrypts the secret key of the SNA node (5). The encrypted message is enclosed by the encrypted secret key and digital certificates of the SNA node (6). The sink node performs data aggregation and, in the same way as the SNA node, performs protection of messages to be delivered to the data processing application via the internet. In this way, sensor node data are additionally protected in the process of transmission via the internet, and securely prove the identity of the SkN node.

In the control center, firstly, de-aggregation of the message is performed (7), and then verification of the digital certificate of SNA node (8). Then, the decryption of the secret key is performed by the RSA algorithm and private key of data gathering application (9). The message is decrypted by the AES algorithm and secret key (10). The digital signature obtained in this way is then decrypted by the public key of the SNA node from the digital certificate accompanying the message. Simultaneously, the message goes through a hash transformation as with the sender, SNA node. Then, obtained hash values are compared (11), and if the comparison is all right, then the message integrity, authenticity, and non-repudiation are preserved. If contrary, the message was modified in the process of transmission or was sent by somebody else.

When verifying the content by the receiver, it is necessary to verify the trust path (if the sender’s certificate can be trusted), duration, and validity of certificates by checking the list of revoked certificates issued by the certification body. If the verification was not successfully completed, then data are not taken into consideration as they were sent by unauthorized or revoked sensor nodes.

When data processing is completed, the management application forwards the management message to actuators A1 (for irrigation) and A2 (for nutrition) in the same way as described above, with the exception that now the management applications is the sender, while actuators are receivers.

## 5. Simulations Results and Proposed Model Evaluation

### 5.1. Performance Evaluation Using LoRa Based WSNs

In this section, the performance of the wireless sensor network with the proposed security key model is evaluated under short and long-range scenarios using LoRa and TelosB based WSNs, respectively.

The LoRa is a long-range technology that provides effective communication of small data with low power consumption. The technology is implemented in the ISM band (the unlicensed radio spectrum reserved for industrial, scientific and medical (ISM) purposes). By LoRa technology, even resource-constrained small sensors or actuators, are able to send data up to tens of kilometers with a network lifetime up to several years even without a power source [49].

As mentioned earlier, private key and secret key are being generated in the sender packet (message), by cryptographic algorithms. In the study by P. Girard [50], it was a marked issue with the LoraWAN key provisioning method, since the network server generates both session keys. This approach could cause a conflict between the priorities of the network server and the application server. The proposed solution of Girard et al. [50] is a new LoRa WSN, which involves the third trusted party. Usually, the end node must use secret keys for a lifetime without updating. Thus, if the key is stolen by the attacker, the intruder can steal all of the data that the target node had previously transmitted [51]. On the contrary, in our scheme, the end node can update the keys in any case. In this paper, the authors propose two rings of network security by encryption and public key for a decryption secret key on the server side. The proposed model of network protection in Section 4 was implemented in a simplified version of the application layer of a sensor node and LoRa gateway in OMNeT++ software. Message data application was coded to consist of key, which is being filtered by the application layer of the LoRa gateway (controller).

**Figure 6:**
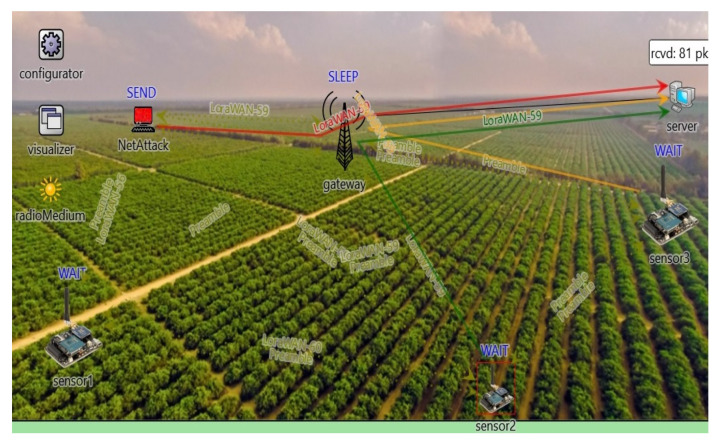
The topology of wireless sensor networks (WSN) for monitoring of agriculture conditions.

**Figure 7:**
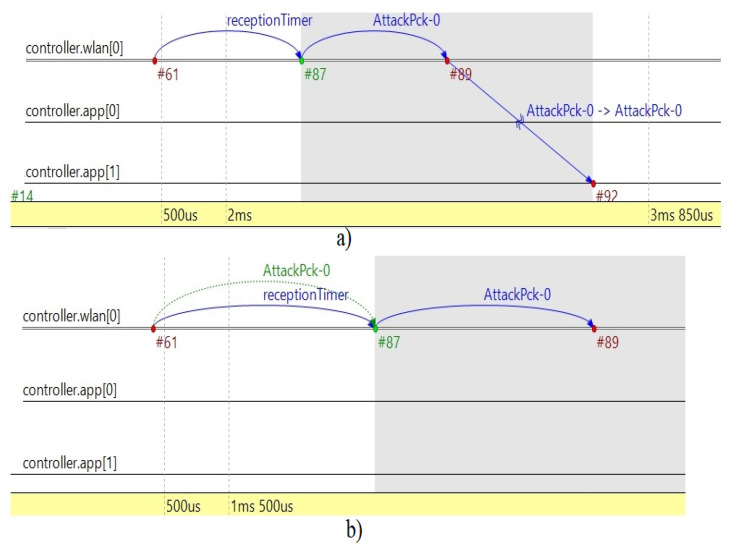
Timeline of simulated transmission of attack packet in wireless (Long Range, LoRa) communication: (**a**) transmitted to the server via gateway application; (**b**) rejected by gateway application with security model.

The network topology of the simulation methodology we used is a modular architecture that relies on the following elements of the LoRa network: three sensors nodes, one sender of attack packet, LoRa gateway, and LoRa server in simulation module Flora in OMNeT++, as shown in Figure 6. The moment when sender NetAttack is transmitting data (attack packet) through the LoRa gateway to the server is described in Figure 6. When the proposed model of the LoRa gateway is implemented in a network topology of the Flora module, the attacker data is being rejected by the application layer (transmitter) of the gateway, controller app [1], see Figure 7b.

If the implemented code is being removed from the application layer of the gateway, attacker data (AttackPck-0) is being received by the application layer of the gateway and transmitted to the server, as shown in Figure 7a. Simulation of key updating is done by the scenario manager, which is updating the modules of the node application and message of the packet (8 bits word) every 20 min.

Modulation of LoRa technology is implemented based on chirp spread spectrum methodology. More specifically, every LoRa symbol is determined by 2 spreading factor chirps, where SF (spreading factor) declares the corresponding spreading factor [52].

The six orthogonal SFs (from 7 to 12) is being used to provide different Data Rates (DRs). In order to avoid issues regarding drift of the crystal reference oscillator, (related to packet delivery) a low data rate optimization mechanism is applied that increase robustness to frequency variation over the timescale of the LoRa message. This can be achieved for SF = 11 and SF = 12. According to the study, with SF = 12, the sensor node requires 10 dBm to transmit data for 5 km in an urban area with minor obstacles. For the simulated scenario, the value of SF = 12 was selected as appropriate for the rural environment. The power consumption of LoRa SX1272 with corresponding transmission powers of the node is presented in Table 2. The modeling of energy consumption was done based on the energy model in Flora. Energy consumption modeling is based on different radio’s states with varying values of power consumption for idle, busy, switching, sleeping, transmitting, and receiving mode of radio. It is assumed that for the proposed LoRa network, the transmission power of 13 dBm is appropriate with CR = 6 (coding rate). Accordingly, the power consumption of the transmitter is adjusted to 92.5 mW in the energy model of the wireless network, for the scenario without the implementation of the security model. Simulated LoRa packet is 51 bytes with assumption of 200 µJ per bit consumed energy.

**Figure 8:**
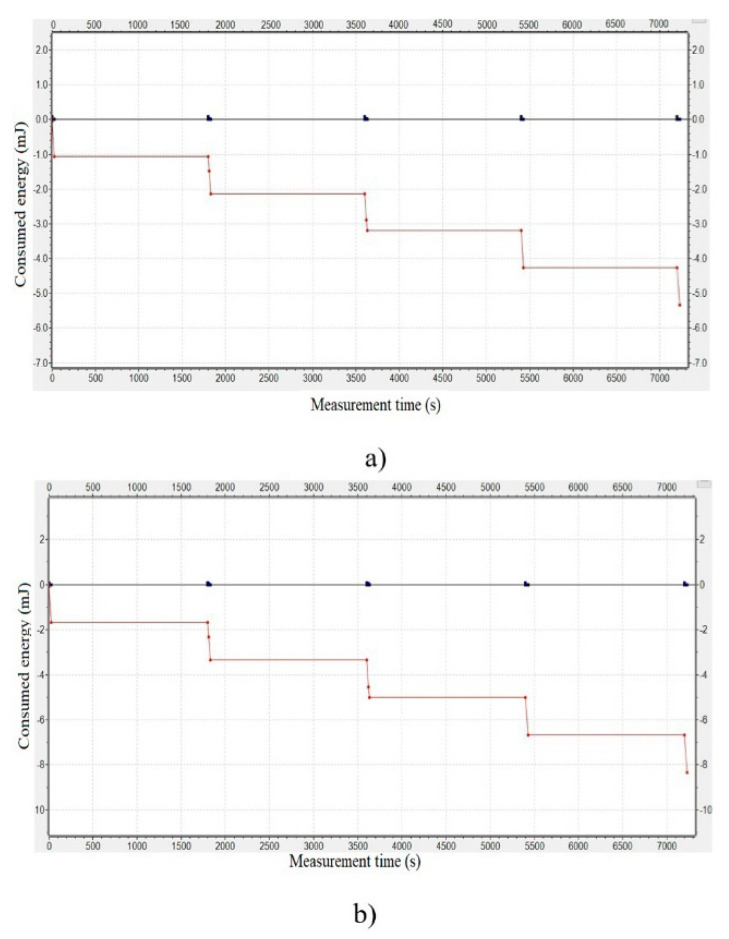
Energy consumption of wireless (LoRa) communication sensor node: (**a**) simulation result without security model; (**b**) simulation result with the proposed security model.

Figure 8a presents the scenario of data monitoring with a simulation time of 2 h (7200 s). Apparently, it is a monitoring scenario with a sleeping period of 30 min (1800 s), which generates two data packets/hour. The sensor node realizes measurements of the agriculture conditions and transmits data every 30 min for the sending period of 25 s. Simulation results showed energy consumption of 1.05 mJ at the sensor node for 92.5 mW of transmitter consumption, Figure 8a.

In order to perform the communication for the attack prevention process, model of sensor and gateway nodes require more energy due to increased bits for transmission. Moreover, the secret key must be updated for every new session, with transmission acknowledgment from the server. Our model is implemented as an additional module within the application of gateway node, without modeling of acknowledgment transmission from the server. Therefore, modeling of consumption for the sensor node, with the proposed security key model, was done by additionally increasing consumption of transmitter “receiving state” for 30%.

As a result, the consumed energy of the sensor node increased by 0.7 mJ for 25 s sending period. According to the results of the performed simulation, energy consumption is 1.75 mJ of one sending period, as shown in Figure 8b. For additional analysis, a simulation on the delivery packet for the different spreading factor was carried out. Due to increased transmission data, which, causing the additional delay on LoRa gateway, packet loss rate (PLR) for SF = 10 was 11/15 for the case, when all three sensor nodes and NetAttack nodes, see Figure 7, were sending packets. For SF = 12, all 15 packets from three sensor nodes during 2 h of simulated monitoring were successfully received at the server. It could mean that optimization of the network model could be done as a future study focusing on delay (waiting time) of LoRa gateway. The effect of the attack prevention process of the proposed model in the wireless network should be optimized further with the main objective to achieve (smaller) more effective SF and packet delivery rate.

### 5.2. Performance Evaluation Using TelosB Based WSNs

The performance of the proposed model has been evaluated using low power TelosB motes developed by University of California, Berkeley [53] in the Castalia simulator [54]. We also used CC2420 radio parameters, which are commercially available, as well as extensively used in sensor applications [55].

The simulation scenario consists of two sender nodes and one receiver, which are located positioned in a square area of 30 m × 30 m. The CC2420 radio module parameters and TelosB node are used. The CC2420 radio has four states: sleep, transmission, reception, and idle listening, which consume the power of 1.4 mW, 57.42 mW, 62.04 mW, and 62.04 mW, respectively. In the network, the sender node sends the data packet of 50 bytes at the rate of 1 packet/s. In the simulation, we assumed digital signature and the encryption key of 32 Bytes, and 33 Bytes, respectively.

WSNs support small size data packets in the network [56]. Therefore, we have used the same data packets size of 50 bytes in the performance evaluation [57]. Each sender node sends the data packet of 50 bytes at the rate of 1 packet/s in the network.

#### 5.2.1. Receiver Energy Consumption Analysis

In order to observe the energy consumption of the proposed model due to overhead added for data authentication, we performed simulations with data authentication that shows each message (data packet) sent with its own signature and encrypted key. Then, results have been compared with no-authentication data transmission scenario, called noDataSecurity. In this scenario, we do not implement data authentication, where each sender node transmits only its data packet without considering data security in the network.

**Figure 9:**
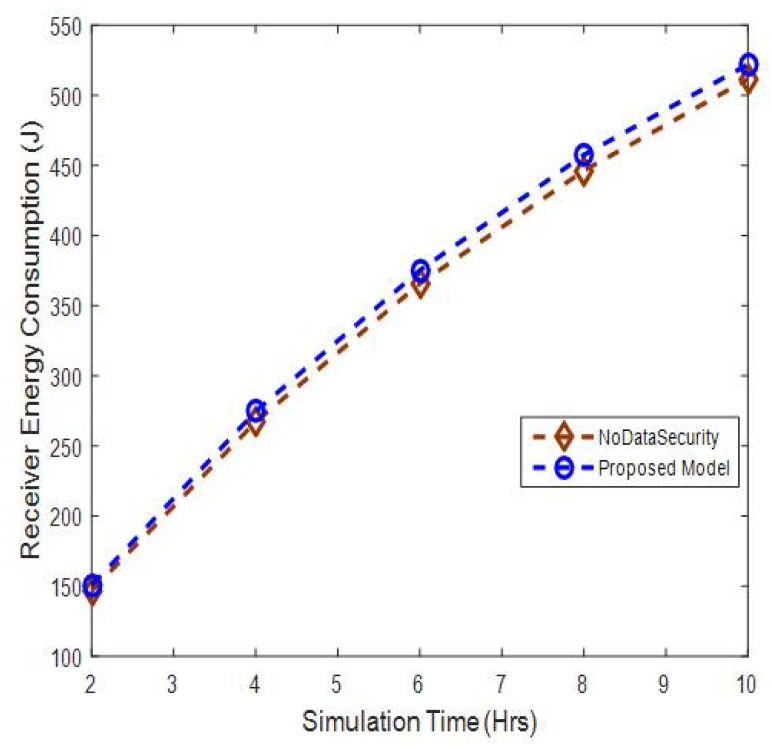
Receiver energy consumption versus simulation time.

The receiver in the proposed model consumes slightly more energy, of up to 2%, when compared with no-authentication data transmission scenario, as shown in Figure 9. This is because the proposed model supports authentication by sending its signature and encrypted key along with its data packet, which consumes energy.

#### 5.2.2. Energy Consumption per Bit Analysis

**Figure 10:**
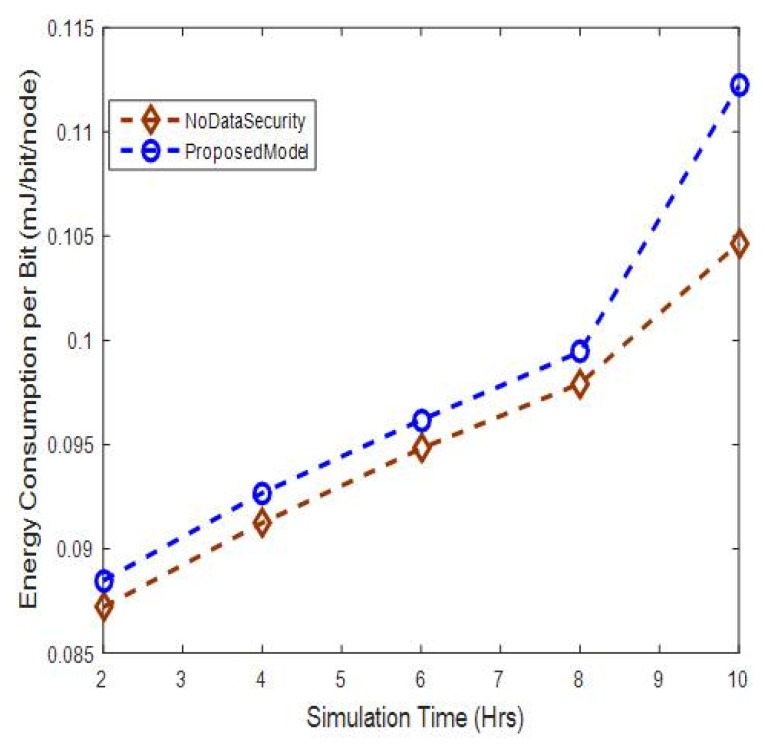
Energy consumption per bit versus simulation time.

The energy consumption per bit is shown in Figure 10. It can be seen that the energy consumption per bit increases slightly up to 1.3% when compared with no-authentication data transmission scenario. It addition, it increases linearly across various simulation times. This reason is that the sender nodes transmit a greater number of data packets when the simulation time increases, which consumes more energy.

#### 5.2.3. Energy Consumption per Sender Node Analysis

**Figure 11:**
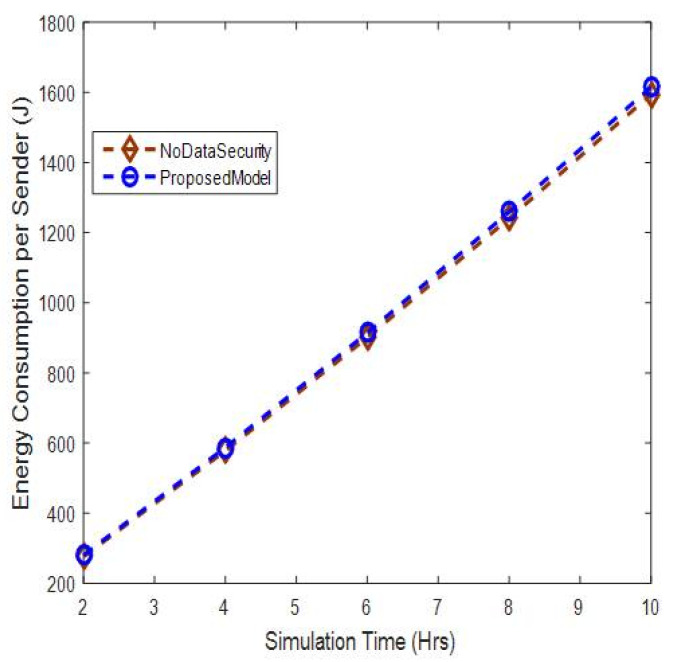
Energy consumption per sender versus simulation time.

The energy consumption per sender node is shown in Figure 11. The results show that the sender node in the proposed model consumes slightly more energy, of up to 7%, when compared to other models without authentication. This is because the proposed model sends a data packet with its signature and encrypted key, which requires more energy in transmission.

## 6. Optimized Model

In the proposed model, each time when the message is sent, the node generates the new secret key for trustworthiness protection. Implementation of such a model requires increased processor strength and memory in nodes, as well as more energy. Resource use in the wireless sensor network with the implemented model depends, also, on selected parameters in the preparation phase for model implementation. This is primarily important for selected key lengths and algorithms, estimated risk, frequency of data sending and management messages. In order to make the model most possible optimized, the authors recommend modifications in hardware and software architecture of the sensor node (technical aspect) and a set of organizational measures (organizational aspect).

**Figure 12:**
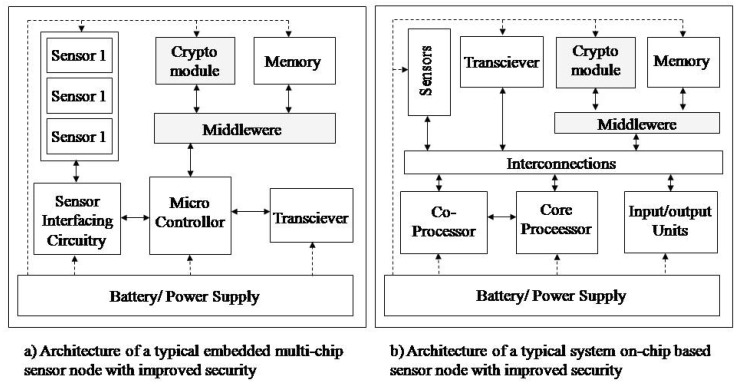
Extended hardware and software architecture of the sensor node.

The technical aspect of model optimization considers modifications of hardware and software node architecture. Therefore, in order to apply the proposed model, it is necessary to expand the existing hardware architecture of the sensor node by crypto-modules, while the software should be expanded by middleware, Figure 12. Crypto-module performs crypto functions (encryption, decryption) and provides a secure environment for key safeguarding. The crypto-module has the following functionalities: generating a pair of keys with an asymmetric cryptographic algorithm (private and public key) and generating a symmetric key with a symmetric cryptographic algorithm (secret key), encrypting a secret key with a public key, decrypting a protected secret key with a private key, encryption and decryption messages, digital signing, onboard secure cryptographic key storage, and key management. The application of the TNODE5 microcontroller for sensors was tested in the paper [58]. TNODE5 is based on a 16-bit microcontroller compatible with the TI MSP430x family. In addition, it has hardware implementation of symmetric and asymmetric cryptography that provides several orders of magnitude improvement in power efficiency over compatible software implementations on commonly used sensor node platforms based on the same type of microcontroller. However, the crypto components of this microcontroller do not have onboard secure cryptographic key storage and key management functionality. Middleware is a software for secure communication of cryptomodules with the operational system of the node.

The organizational aspect includes a set of organizational measures in the planning process, preparations for implementation and implementation of sensor wireless networks in agriculture aiming at the optimization of the proposed model.

In addition to modifications in hardware and software architecture of the sensor node, it is necessary to implement optimization by imposing a set of appropriate measures for organizing the application of the model in the sensor network.

Organizational measures to be implemented during the organization of application of the model:

- Crypto-parameters planning. Based on the security risk assessment of the wireless sensor network, the needs assessment for cryptology algorithm, hash functions, and key lengths to be applied are conducted. Lesser values of key lengths, as well as simple crypto-algorithms create less burden for the processor, bringing the faster response and smaller energy use. Larger values of key lengths should be used in the nodes for data aggregation as they have increased hardware capacities, but, also, increased risk from the attack as they process the increased amount of data.
- Secret key use planning. The proposed model has a new secret key generated for each message, and then the key is encrypted and transmitted together with the sender to the receiver. This use of the secret key brings to the establishment of strong trustworthiness between the sensor node and the message receiver. However, this manner of secret key use uses the processor and energy more than permanent or temporary secret keys are used. This key is periodically replaced in all nodes and is used for message protection until his time-lapse. Now, there is no need to generate a new key for each message and thus spend additional hardware resources and time for its protection.
- Data gathering planning. A sensor node may gather more types of data. Sending of all kinds of data or any specific data brings to more frequent encryption and resource utilization of the node than when the transmission, and encryption of all node data gathered simultaneously is performed. Data gathering scheme should be planned, taking into consideration the use of all resources caused by frequent encryption.
- Certificate validation and verification planning. During digital signing and development of digital envelope, it is necessary to perform verification of the certificate validity, meaning it is necessary to verify if the certificates in the trust chain are revoked or suspended. In order to perform this verification, it is necessary for the node to have access to the list of revoked certificates, or a possibility to send an inquiry to the certification authority on the certificate status. In both cases, transmission capacities and network nodes are additionally burdened. Authors recommend conducting verification of the digital certificate at the receiver’s (aggregation node, processing application) as it has increased communications and hardware capacities.
- Communications network architecture planning. In a sensor network, nodes can transmit data to the aggregation node by transmitting it from one to another node. If the model is applied to the communications of the two neighboring nodes, then there is an additional burdening of nodes unlike in the case if only nodes transmit the protected message. In order to escape additional node burdening, it is necessary to realize protected communications between the sender’s node and receipt location. This may be a node aggregating data of more nodes by some criteria or end application for data processing. This is why it is necessary to organize sensor network communications from the sender’s node to the receiver’s location.

**Figure 13:**
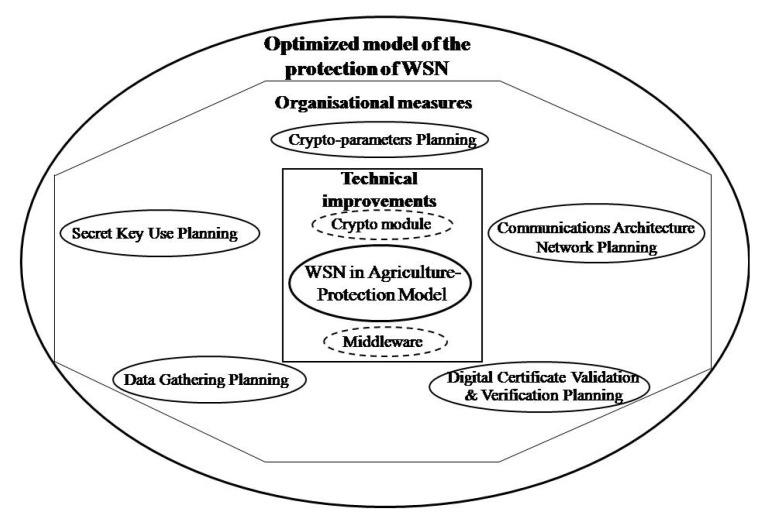
Optimized model of the protection of sensor networks in agriculture.

Upon completion of the technical and organizational optimization of the proposed model, the optimized model was developed, as shown in Figure 13.

## 7. Conclusions

Currently, WSNs have gained significant attention in agriculture. The analysis of gathered data from the farming field plays an important role in agricultural cultivation. However, malicious competitors can add or modify measured data and disrupt the application and process of production or testing. Therefore, this paper proposed the data security model, which enables data protection from the sensor via the communications network to management structures, and vice versa. The simulation results showed the efficiency of the model in preventing attacks during communication, but also an increase in energy consumption. Frequent change of the secret key causes the highest energy consumption, while the sender and receiver energy consumption is slightly higher. The proposed optimized model of WSN data protection includes a set of technical and organizational measures in order to speed up the data protection process and use, rationally, sensor hardware resources.

Except for being applicable in WSN in agriculture, the model may be applied in any field using sensors, WSN, requiring a high level of data security.

## Figures and Tables

**Table 1:** Parameters for the model initialization.

Initialization Parameters	Parameter Value	Security Requirements
Symmetric algorithm AES	Key length 128 bits	Message trustworthiness and privacy
Asymmetric algorithm RSA	Key length 1024 bits	Secret key trustworthiness and privacy and message integrity
Message-digest algorithm SHA-1	Message-digest length 128 bits	Message integrity
Digital certificate	1024 bits key length and SHA-1	Communication party authenticity, as well as non-repudiation of message reception

**Table 2:** LoRa SX1272 characteristics.

Transmission Power (dBm)	Power Consumption (mW)
20	412.5
17	297
13	92.4
7	95.4
